# Testing personality—pace-of-life associations via artificial selection: insights from selected lines of rainbow trout on the context-dependence of correlations

**DOI:** 10.1098/rsbl.2024.0181

**Published:** 2024-06-19

**Authors:** Peter A. Biro

**Affiliations:** School of Life and Environmental Science, Deakin University, Geelong 3216, Australia

**Keywords:** personality, pace-of-life, artificial selection

## Abstract

More than a decade of study since the personality pace-of-life syndrome (POLS) hypotheses were first proposed, there is little support for it within species. Lack of experimental control, insufficient sampling in the face of highly labile behavioural and metabolic traits, and context dependency of trait correlations are suggested as reasons. Here, I argue that artificial selection and/or use of existing selected lines represents a powerful but under-used approach to furthering our understanding of the POLS. To illustrate this potential, I conducted a focussed review of studies that compared the behaviour, metabolism, growth and survival of an artificially selected fast-growing rainbow trout relative to wild unselected strains, under varying food and risk conditions in the laboratory and field. Resting metabolic rate, food intake, and behaviours that enhance feeding but increase energy expenditure (activity, aggression, boldness), were all higher in the fast strain in paired contrasts, under all food and risk conditions, both in the laboratory and the field. Fast-strain fish grew faster in almost every food and risk situation except where food was highly limited (or absent), had higher survival under low or zero predation risk, but had lower survival under high risk. Several other traits rarely considered in POLS studies were also higher in the fast strain, including maximum swimming speed, and hormones (growth hormone (GH), thyroid hormone (T3) and insulin-like growth factor (IGF-1)). I conclude: (i) assumptions and predictions of the POLS hypothesis are well supported, and (ii) context-dependency was largely absent, but when present revealed trade-offs between food acquisition and predation risk. This focused review highlights the potential of artificial selection in testing POLS ideas, and will hopefully motivate further studies using other animals.

## 1. Introduction

Nearly two decades ago, behavioural ecologists began to focus their attention on animal ‘personality’ for the first time [1]. This represented a paradigm shift for behavioural ecologists at that time, from a view of behaviour as being completely flexible, to one of limited flexibility. This shift in thinking subsequently sparked many hypotheses on the proximate constraints and ultimate consequences of animal personality (defined as individual differences in behaviour that are consistent over time and/or contexts).

One of the most influential hypotheses proposed to understand animal personality centres around the integration of metabolic, behavioural and life-history traits. These hypotheses predict individuals should exhibit consistent individual differences in metabolic rate, behaviour and life-history productivity, and that these traits should be mostly positively correlated among individuals [2–6], but see [7]. Placed into statistical terms, it proposes that the traits have substantial repeatability, and significant positive trait covariance, of the individual predicted (mean) values for each trait. Hormones that integrate and coordinate trait associations are, of course, also implicated [6], but seemingly few studies have incorporated them into study designs. The integration of metabolic, behavioural and life-history traits along a slow to fast continuum was labelled the ‘pace-of-life syndrome’ (POLS) hypothesis [6]. Although the POLS hypothesis is labelled as if it were a single hypothesis and the acronym widely adopted, as outlined above it is actually a collection of highly related ideas, with many overlapping predictions and assumptions, but where some predictions are rather different from each other (discussed below).

At its core, the POLS hypothesis explicitly assumes that higher resting metabolic rate (RMR) is an index of the capacity to acquire, process and output energy on a sustained basis, whereby higher sustained metabolism fuels and is fuelled by behaviours that acquire energy and/or consume energy, and fuels the production of new biomass [3,5]. This assumption has been referred to as the ‘performance’ model of energy allocation [4], and consistent with this explicit assumption is that, in the case of growing or reproductive individuals, consistently productive individuals should have high resting metabolism and behaviours that support high consumption such as high levels of activity, aggressiveness, and boldness [3]. The assumption that individuals consistently differ in productivity is evidenced by the vast literature on successful artificial selection on productivity in agriculture. Less clear is the role of RMR, however RMR is likely to be an index of the capacity for sustained energy output, as evidenced by the prevalence of positive correlations between RMR and measures of sustained energy output like daily energy expenditure (DEE); [8], and given that RMR is often correlated with the size and activity of energetically expensive organs that support high intake and energy metabolism, including the intestines, liver, kidney and heart (reviewed by [5,9]; also this review).

Mechanisms that might maintain variation in behaviour, metabolism and life history most likely involve cost-benefit trade-offs between slow and fast strategies. Such trade-offs may include behaviorally mediated trade-offs between productivity and mortality owing to predation on risky behaviours [2,3,5], and negative impacts of high metabolism on body condition and growth when food is scarce [5,10,11]. Thus, we expect consistently productive individuals to incur high mortality, and suffer the impacts of food scarcity more than unproductive individuals. Another trade-off that can maintain variation in behaviour involves a trade-off between early and late life reproduction [6,7]. However, this trade-off assumes productivity does not consistently differ among individuals, whereby risk-prone individuals reproduce early at the expense of future reproduction, and that all individuals survive through the reproductive intervals considered [7]. Thus, productivity in that model is not consistently correlated with behaviour through time. By contrast, other theory assumes that individuals differ in ability to acquire and metabolize energy and thus behaviours that increase intake rate should be consistently and positively correlated with productivity through time [2,3,5].

## 2. Current state of evidence for the pace-of-life syndrome ideas

Since those POLS ideas were presented over a decade ago, there have been many empirical tests of the hypotheses, but few studies have found compelling evidence (e.g. [12–14]). Indeed, recent meta-analyses reveal little to no evidence for the POLS predictions at the among individual level ([15–17] but see [18]). Reasons why most studies reveal very weak or no correlations may be that correlations are variable and thus ‘context dependent’ [10], studies are generally poorly sampled and estimate parameters with poor precision and thus downwardly bias correlations [15,19,20], and/or the POLS hypothesis may need to be re-evaluated [15,16]. By contrast, a key assumption of the POLS hypothesis that higher RMR supports greater sustained energy expenditure and upregulation of energy expenditure [3,5] is supported by reviews and meta-analysis: these show that RMR is positively correlated with DEE and with ability to upregulate energetic output when needed ([8,18,21] see also [22]).

## 3. Testing the pace-of-life syndrome is challenging

Testing relationships between metabolism, life history and behaviour at the among individual level is difficult. Powerful tests require either controlled conditions or conditions that can be well quantified and accounted for statistically and require large sample sizes in terms of number of individuals and terms of repeated measures per individual [3,5,12]. Large sample sizes are required for traits like behaviour and metabolism, particularly at the within individual level (i.e. repeated measures), because behaviour and metabolism are labile traits with rather low repeatability [23–25]. Simply put, this means that the ‘signal' (an individuals’ mean) is low relative to residual ‘noise’, and large sample sizes are needed to estimate them with precision [20]. Furthermore, individuals are also likely to differ substantially in residual variation [26], meaning that some individuals' mean can be estimated precisely with just a few repeated measures, while others cannot.

## 4. Artificial selection as a powerful means to test the pace-of-life syndrome

By contrast to an among-individual focus on these questions, an under-used but extremely powerful approach to study the POLS hypothesis is via artificial selection. Artificial selection can greatly increase available trait variance for study, allowing us to test whether other traits are functionally and/or genetically correlated with it [27,28]. This approach effectively provides a stronger means to find the signal among the great level of noise that is inherent in studying labile traits like metabolism and behaviour. In support of this approach, early reviews found support for the POLS hypothesis, whereby many of the reviewed studies compared traits across selected lines of animals, including livestock ([5] see also [3,29]). For example, lines of mice selected for high RMR also show higher food intake, higher activity rates, and greater juvenile growth rates than the low RMR lines [30,31]. Similarly, selection on boldness has resulted in lines of bold junglefowl with higher RMR, higher food intake, higher food conversion efficiency, larger eggs, and faster growth rates [32,33].

Here, I review and summarize a number of studies comparing the among-strain differences in metabolism, behaviour, growth and survival between a strain of rainbow trout selected for high intrinsic growth rate and unselected wild strains. These traits were measured under varying conditions of food abundance and predation risk conditions, both in the laboratory and in the field, and for two size/age classes. This permitted an assessment of the context-dependence of trait correlations across strains; that is, if growth is faster in the ‘fast’ strain under abundant food, but not scarce food, but behaviours are always more bold in the fast strain, then we can conclude context-dependent correlations where growth and behaviour are correlated under high, but not low food. Although these studies were not designed to test the POLS predictions, they each bear directly on these predictions, and when their collective results are brought together in one place they provide compelling insights—they illustrate trait associations and the context dependency of trait associations (if any), and in doing so reveal selective pressures that might contribute to maintenance of trait variation. This targeted mini-review thus serves as an inspirational one to highlight the unrealised potential of artificial selection and/or use of selected lines to study POLS ideas, hopefully inspiring other more extensive reviews and studies using existing lines.

## 5. Literature review outline

Using ISI Web of Knowledge I searched the literature search for studies that made use of a ‘domestic rainbow trout’ strain, specifically one that has been selected for high intrinsic growth rate under hatchery conditions. This is distinguished from many other studies that use unselected strains in a hatchery environment that are often referred to as ‘domestic’ or domesticated. I then viewed the abstracts and/or methods to ensure that the study made direct comparisons of at least one of either metabolism, behaviour or life-history traits of the domestic relative to a wild strain when exposed to identical conditions. Once relevant articles were located, I then additionally searched the citing articles of each one for the words ‘rainbow trout’ to potentially identify additional articles missed in the initial search.

Most of the studies I summarize in this synthesis paper made use of a single strain of trout which has undergone selection for high intrinsic growth rate, often referred to as the ‘Fraser Valley domestic’ (FV) rainbow trout. This strain was created by artificial selection on growth rate in a hatchery environment, with the aim for use in stocked populations to provide rapidly growing and large fish for anglers; it resides entirely within the hatchery system that originated from a single stock in California, USA, that was subsequently distributed to the Fraser Valley Trout Hatchery, and then later on was distributed to other nearby private hatcheries in British Columbia, and Ontario, Canada (K. Scheer, Operations Manager, Fraser Valley Trout Hatchery, BC Ministry of Environment 2003, personal communication; [34]). It grows about twice as fast as wild strains raised similarly in the hatchery under ad libitum food conditions (this review).

A handful of additional studies identified in this review used strains of trout which may or may not be closely related to the FV domestic, named the Arlee and Hot Creek domestic strains, and those studies compared their performance to nearby wild-type strains in the USA. One study was included for its information on growth, but its estimation of ‘routine metabolic rate’ was not used as this measure of metabolism has no clear interpretation in the context of the POLS hypothesis; triploided (3n) fish were not considered, and only data on unmanipulated diploid (2n) wild and 2n FV domestic fish were used in this review [35]. Another study used a 3n domestic strain contrasted to a wild one, and this one too was excluded but it is noteworthy that the domestic strain had higher RMR [36]. One study, included in this review, used a domestic strain with origins from a combination of FV domestic and similar high growth-selected strains from California, USA [37].

## 6. What do selected lines of trout tell us about pace-of-life syndrome?

A total of 12 different studies were identified and subsequently summarized which used the up-selected ‘fast’ strain and contrasted its performance (in terms of metabolism, and/or behaviour and/or life history) with a wild-type strain(s) also raised in the hatchery; in addition, several more studies were identified that quantified other traits, peripherally related to the current POLS ideas, and are presented below in the Discussion. In every one of the reviewed studies, the fast strain performance was contrasted to one (or more) slow strains of unselected wild trout raised identically in the hatchery. The wild strains were obtained by collecting eggs and sperm from the source population in the wild and then raised in the hatchery alongside the fast strain under identical conditions and fed ad libitum; the fast strain was raised from gametes collected in the hatchery.

Overall, the studies could be grouped into those that quantified differences between strains under laboratory conditions (without any predation risk, nine papers; table 1), and those doing the same under semi-natural stream and natural lake conditions where predation risk varied from low to high (five papers; table 2). Where possible, I have provided approximate effect sizes of strain differences in the main text, as inferred from graphs showing means of each strain, as strain differences and uncertainty estimates were not always provided.

**Table 1.** Trait differences between strains in the laboratory, in relation to food availability. (Summary of studies (one per row), comparing trait performance of an up-selected ‘fast’ growth strain relative to an unselected wild ‘slow’ strain of rainbow trout, in relation to food level. An entry of ‘+’ indicates significantly greater trait values in the fast relative to the wild strain, a ‘–’ indicates significantly lower values, ‘nil’ indicates no significant difference, and ‘na’ or blank cells indicate that trait was not measured. In one instance (6), no statistical analysis was performed but the difference was very substantial and retained in the table as if significant.)

metabolism			behaviour			energy acquisition		life history			source
RMR			aggression activity		boldness	food intake	feed conversion	growth			
high food	medium	no food	high food	high food	high food	high food	high food /med.	high food	medium	no food	
+	+	+				+	+/nil	+	+	nil	[38]
						+	+/na	+			[39]
						+	+/na	+	+		[37]
								+			[40]
								+	+		[41]
				+	+						[42]
			+					+			[35]
								+			[43]
								+			[44]

**Table 2.** Trait differences between strains, and how it varies in relation to predation risk. (Summary of studies comparing behaviour, growth and survival of an up-selected ’fast’ growth strain relative to an unselected wild ‘slow’ strain of rainbow trout, in relation to predation risk. Symbols indicating higher (+), lower (–) or no significant difference (nil) between the fast and slow growth strains are where it was assessed in each study.)

age and situation		behaviour				life history				source
age class	setting	activity		boldness		growth		survival		
		low risk	high risk	low risk	high risk	low risk	high risk	low risk	high risk	
age-0+	lake	+	+	+	+	++	+	+	−	[39]
age-0+	stream	+	+	+	+	nil	+	+	−	[41]
age-0+	lake						+		+/−	[45]
age-1+	lake	+	+	+	+	nil	+	+	-	[34,46].

### (a) Across-strain trait differences under varying food rations (in laboratory, no predation risk)

Under ad libitum food conditions, the fast strain achieved growth rates about twice that of the slow strain (100% higher) in every case where it was assessed (eight studies) in the laboratory (table 1); this confirms the expected large differences in intrinsic growth rate that is strain specific, which is underpinned by additive genetic variation (see Discussion). The high growth rate in the fast strain was achieved principally by substantially higher food consumption but was also aided to a lesser degree by higher growth (conversion) efficiency (table 1). Fast-strain fish were more aggressive in one laboratory study, more active and bold in another (table 1). Activity and boldness were about 1.5 to 2 times higher in the fast strains, depending on time since the last feeding (5) and aggression was *ca* 2 times greater in fast lines (6). Higher RMR in the fast strain (*ca* 20%) was associated with higher consumption (*ca* 40%) and about 2 times higher growth within one study (1). Positive correlations between behaviour and growth rate were evident in two studies (5,6), and positive correlations between behaviour, RMR and growth could be inferred *across* studies more generally as all studies showed the fast strain to have higher values of all traits under ad libitum, and even medium, food levels (table 1).

However, under conditions of food restriction or starvation, growth differences between strains were still present, but were reduced (three studies: 1, 3, 5) and disappeared (one study: 1), respectively (table 1). RMR decreased with ration, but RMR *differences* between the strains were maintained, with the fast strain always having higher RMR at a given ration (study 1; table 1). Thus, RMR and growth rate were positively correlated under most conditions, but absent under starvation conditions within one study (1). Taking inference from comparisons *across* studies, we can conclude no correlations between behaviour, RMR and growth rate under starvation conditions owing to the fast strain losing its growth advantage without food (table 1).

### (b) Across-strain trait differences under varying predation risk (in laboratory and field settings)

In terms of behaviour, the fast strain was more active and bolder than the wild strain it was contrasted with, under all risk conditions (low or high), in both natural lake and semi-natural laboratory settings, and for both small age-0 fish and for much larger (small adult size) age-1 + fish (table 2). The fast strain grew faster in high-risk conditions (in all four studies; 20 to 100% faster), but under low-risk conditions the growth differences were inconsistent: fast strain fish were higher in one study, and there was no difference in two further studies (table 2). Thus, across-strain correlations between activity, boldness and growth rate were all positive under high risk, but positive (one study) or no correlation (two studies) under low risk (table 2). Fast strain fish had higher survival under low-risk conditions (40–100%) but lower survival under high risk (40–60%; studies 1,4). One study found higher survival of the fast strain during the growing season (3), but subsequently experienced lower overwinter survival in those same lakes (table 2). Thus, activity, boldness, and growth were negatively correlated with survival under high predation risk, but positively correlated when risk was low.

## 7. Review supports pace-of-life syndrome predictions

In brief, the POLS hypothesis predicts that individuals or genotypes with high RMR should also be more active, aggressive and bold, have higher food intake rates, and have greater capacity for growth and reproduction. Overall, nearly all the predictions were supported by the reviewed studies. Of course, any support for the POLS ideas is limited to the lines and species considered here, but it does provide a compelling basis from which to motivate further studies on existing selected lines in other species, or to motivate new artificial selection studies to test the ideas and the assumptions.

The reviewed studies showed that RMR, food intake, and behaviours that enhance feeding but increase energy expenditure (activity, aggression, boldness), were all higher in the fast strain in paired contrasts, under all food and risk conditions, both in the laboratory and the field. Fast-strain fish grew faster than slow-strain fish in almost every food and risk situation, except where food was absent or highly limited. Thus, positive correlations between behaviour RMR, behaviour and growth rate across strains were observed in high and medium food levels, but these correlations disappear under highly food-limited situations owing to equal growth between strains in that situation. Fast-strain fish had higher survival under low or zero predation risk in field conditions, but lower survival under high risk, meaning that behaviour, RMR, growth and survival are positively correlated across strains under low risk, but negatively correlated under high risk. Given that for fish in general, and salmonids specifically, growth rate determines fish size at age and fecundity in females is size dependent means that by extension we can infer reproductive benefits of rapid growth in the fast strain as well. The observed trait differences, and trait covariation, were all probably genetically based given that the growth performance of pure strains and F_1_ and F_2_ back-crosses between the fast domestic and slow wild trout strains was consistent with additive genetic variance [40].

## 8. Assumptions of pace-of-life syndrome supported

An important assumption for the POLS predictions to hold is that RMR is a functional correlate of the ability to acquire, process and generate energy on a sustained basis (see Introduction). This was supported by the observation of increased intake rate by the fast strain in every situation it was observed (except starvation conditions) and larger internal organs associated with the metabolism of food into useable energy (stomach and intestine length) that presumably account for the observed increased food conversion efficiency [38,39].

Another assumption of the POLS is that high metabolic costs might weigh disproportionately heavily on individuals or genotypes when food is scarce, that would lead to reduced or no correlation between RMR, behaviour and biomass production [5,10]. This assumption was supported by observations of the fast strain not growing faster than the slow strains under limited or absent food. Yet another assumption of the POLS is that behaviour mediates trade-offs between growth and mortality, whereby high productivity is supported by risky foraging behaviours, representing another mechanism that might help maintain variation in populations [2,3]. This was supported by observations of higher survival rates of the fast strain under low risk, but lower survival rates under high risk. When food and/or risk vary substantially temporally or spatially, this is a means by which variation and covariation of traits might be maintained in the population.

## 9. Context dependence of trait correlations?

Recent studies have suggested that context dependency might often alter trait correlations from those predicted by the POLS, and account for the many studies that find no associations [10,15,16]. Here, I find that across a range of conditions, most correlations are in line with that predicted by the POLS showing relatively little or no context dependence.

First, there was no context dependency of behaviour observed in any of the studies—the fast strain was always more active, aggressive and bold, regardless of risk context. Unfortunately, the laboratory studies mostly quantified behaviour only under high food conditions, and so this review cannot comment on any potential context dependency of behaviour in relation to food abundance in the laboratory. However, in semi-natural stream channels with limited food, and the lake experiments, there was presumably some level of food limitation, and yet the fast strain was always more active and/or bold in those studies.

Second, there was no context dependence of RMR. As expected, RMR decreased with reduced food availability in the laboratory—but it was always higher in the fast strain even under starvation conditions. Higher RMR was associated with higher growth in all conditions, except starvation conditions where growth did not differ between strains, thus meaning an absence of an RMR-growth relationship among strains. This is somewhat surprising, as we might expect that a high RMR in the absence of food might result in lower growth rates, which has been proposed as a cost of having elevated energetic capacity [5,10]. However, the fast strain was larger and thus had higher lipid content prior to removal of food, and this might explain why there was no mass loss under starvation if fat content was replaced with water as it was metabolized. Indeed, the fast strain did have higher water content under starvation conditions [38]. Thus, the fast strain probably experiences greater losses of energy stores relative to wild strains owing to its consistently higher RMR.

Third, context dependence of trait associations was apparent across conditions of varying predation risk. Survival of fast trout was higher under low or zero predation risk, but was lower under high risk of predation and is lower than slow strains in most studies. This result is not surprising when higher growth rate is associated with elevated activity and boldness as it often is [47–49], and it is an assumption that has been discussed in the context of trait covariance along a slow-to-fast continuum [1–3].

## 10. Extending pace-of-life syndrome to include performance and ‘other’ traits?

In the course of reviewing the literature, I located several studies that provided information on hormones, performance traits and even brain size that are not a presently a focus of the POLS hypothesis, but worth noting here. One study found that the fast strain had higher levels of hormones related to growth, including growth hormone (GH), thyroid hormone (T3) and insulin-like growth factor (IGF-1) when held under ideal laboratory conditions with ad libitum food [50]. This is an interesting and expected result given that one would expect the endocrine system to coordinate the integration of metabolic, behavioural and life-history traits, and given the well-known links between those hormones and somatic growth. Indeed, the endocrine system is discussed explicitly in at least two of the original conceptual papers developing the POLS ideas [4,6], although it seems rarely discussed or studied presently.

Other studies revealed differences in ‘performance’ traits between the fast and slow strains of trout, consistent with theory about how and why these traits might also logically be part of the POLS hypothesis [51]. Three studies each found that the fast strain had higher maximum swimming speed (termed ‘U-crit’) than slow strains when challenged in swim tunnels [35,43,44], and one study found that the fast strain had lower maximum metabolic rate (MMR) when fish were fed ad libitum in the laboratory [38]. This last result is somewhat surprising given that correlations between RMR and MMR across individuals or genotypes is expected when they share similar organs and organ systems and where higher RMR is a means to pay back high oxygen deficit costs of maximal output [51]. Finally, one study revealed that the size-corrected masses of the stomach and intestines were greater in the fast strain relative to several slow strains [38]. This result is expected when intake rates are consistently high alongside rapid growth rates and higher levels of behavioural activity, and these organs support high intake rates and subsequent high energy output. In fact, these organs (among others) account for a large portion of the total resting energy output when not digesting food.

The higher U-crit observed in the fast strain indicates that high RMR is associated with measures of aerobic swimming performance and capacity. It has been suggested, and some reviewed studies indicate, that these associations exist owing to their functional connection to organs and systems that support one another while active and at rest [9,51]. Indeed, Auer *et al*. [11] observed a positive correlation between maximum ration and MMR in juvenile brown trout, and Norin & Malte [52] found a positive correlation between RMR and MMR in individual brown trout. Thus, it is odd that higher MMR was not observed in the fast strain in one study when several others show U-crit is higher, especially since they are measured on the basis of similar swimming challenge tests.

Links between personality and cognition have been proposed, and in general results are equivocal where on average there is no relationship, except that bolder individuals may be faster learners but only under risk of predation [53]. In this review, two papers were identified which measured brain size differences between fast and slow strains of trout. Two studies found smaller brain size and lower gene activity in the brain in fast strain trout [54,55], whereas another study found evidence suggesting the opposite [42]. Further studies might help reveal what the general pattern between these strains is, if there even is one.

## 11. Limitations of strain comparisons

One could argue that the use of the inbred and highly selected domestic strain, which resides only within the hatchery system, may have limited relevance when compared to wild strains obtained recently from the field. For example, it is possible that the fast strain has lost some behavioural attributes, including a loss of antipredator behaviour owing to predator absence. However, evidence indicates the ‘fast’ domestic strain does indeed show appropriate responses to predators. For example, it still displays normal antipredator behaviour in the laboratory and in the field, showing substantially reduced activity and reduced use of risky habitats under predation risk [34,39,41,46].

An implicit assumption I make here is that results are comparable across studies, owing to the use of the same fast (domestic) strain, and wild strains that are assumed to be very similar in terms of the traits considered here. Several studies used identical strains for comparisons (FV domestic versus Pennask wild strains for example), but some compared the fast to other wild strains. All indications are that differences among wild strains in terms of metabolism, consumption and growth are negligible [38,42], making comparison of the FV domestic to a given wild strain to be valid and comparable even across studies using different wild strains. A related limitation is the comparison of wild strains to a single selected strain, making inferences about correlated evolution equivocal—we would ideally like to have several selected lines to exclude possibilities of mutation and drift [56].

It is reassuring to note that some of the strain differences noted in the review are also evident within wild populations of this species. Strains of rainbow trout in the wild that achieve large body size have higher metabolic rate, higher intake capacity, higher digestive efficiency, larger digestive organs and bolder behaviour than small-strain fish when assessed under common garden conditions [57]. Further, there is evidence for fishes that RMR is positively correlated with dominance and growth rate more generally, and that this advantage may deteriorate when food conditions decline [58]. Finally, selection on boldness in flatfish reveals that bold line fish grow faster, have higher RMR, higher MMR and thus greater aerobic scope than shy line fish [59].

## 12. Future studies using artificial selection

I suggest that future studies take advantage of existing selected lines of animals for studying trait correlations related to the POLS hypothesis. In the case of the trout example reviewed here, an ideal future study would involve the measurement of RMR, behaviour and growth within a single study where both food and risk are manipulated in a factorial design. Such an study would be made particularly compelling if the design could involve more than two levels of food/risk, as effects may not be linear. In addition to the trout system described here, one could use other existing selected lines of animals by seeking out collaboration with those researchers. Starting ones own selection line experiment to study the POLS is not an easy proposition, but one that is possible and realistic in relatively short time frames if one were to use invertebrate models (e.g. [28]), or vertebrate models with rapid reproduction like the mosquitofish [60].

In conclusion, I believe that this review has provided evidence to support the predictions of the POLS hypothesis, including support for some of the key assumptions underlying it. It highlights the value of using artificial selection and/or existing selected lines as a means by which to obtain samples that differ substantially in some key trait related to the POLS, increasing signal relative to noise, and thus provide a means for a powerful contrast of labile traits expected to covary with one another.

## Data Availability

This article has no additional data.
